# P-2259. Incidence, Treatment, and Outcomes of Urinary Tract Infections Caused by Extended-Spectrum Beta-Lactamase Producing Bacteria in Kidney Transplant Recipients

**DOI:** 10.1093/ofid/ofae631.2412

**Published:** 2025-01-29

**Authors:** Elise N Gerdes, Michael A Wynd, Melissa Chaung

**Affiliations:** The Mount Sinai Hospital, Princeton, New Jersey; Hackensack University Medical Center, Hackensack, New Jersey; Hackensack University Medical Center, Hackensack, New Jersey

## Abstract

**Background:**

Urinary tract infections (UTI) are the most common bacterial infection in kidney transplant recipients (KTR) during the first year post transplant. The objective of this study was to determine the incidence of UTI caused by extended-spectrum beta-lactamase (ESBL) producing bacteria in KTR and compare their outcomes to KTR with UTI caused by non-ESBL producing pathogens.

Index Urinary Tract Infections (2013-2022)

**Figure:**
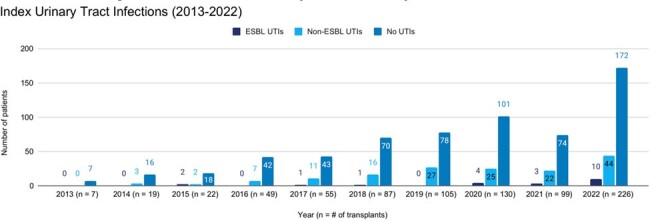

**Methods:**

This was a single-center, retrospective, chart review including adults who received their first, kidney-alone transplant from January 2013 through December 2022 and had a UTI within the first year post-transplant. A UTI was defined as a positive urine culture treated with antibiotics and presence of ESBL-producing bacteria was confirmed by susceptibility patterns. Patients were excluded if they had a rejection or received augmented immunosuppression prior to index UTI, received treatment for positive donor cultures, or were dialysis-dependent at 90 days post-transplant. Primary outcomes: incidence of index UTI caused by ESBL producing bacteria, location of treatment, and length of stay. Key secondary outcomes: patient and allograft survival, incidence of biopsy-proven rejection after treatment of index UTI, and number of UTI in the first year post-transplant.

Primary Outcome: Hospitalizations

**Figure:**
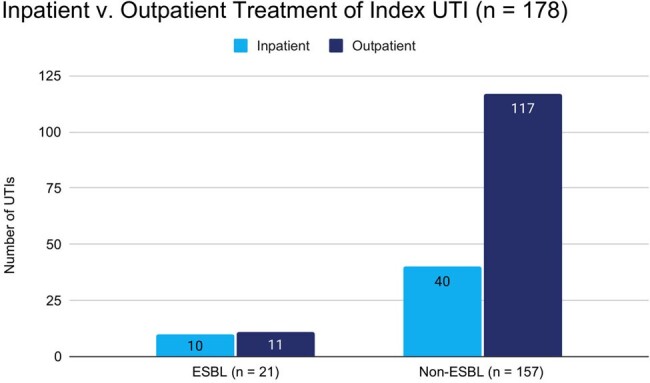

**Results:**

Of 178 patients included, 21 (11.8%) had a UTI due to ESBL-producing bacteria and 157 (88.2%) had a UTI caused by a non-ESBL producing organism. The incidence of ESBL-UTIs increased from 0 to 18.5% during the observation period. No differences were identified in baseline characteristics except for receipt of a donation-after-cardiac-death kidney, 8 (38.1%) in the ESBL-UTI group and 28 (17.8%) in the non-ESBL UTI group, p = 0.03. Compared to the non-ESBL-UTI group, the ESBL-UTI group was more likely to be admitted for treatment 11/21 (52.2%) vs 40/157 (25.5%), p = 0.01, and had more UTIs in the first post-transplant year, mean (range) 2.7 (1-6) vs 1.8 (1-7), p = 0.003. No differences were identified in other outcomes.

Empiric Therapy in Patients with Extended-Spectrum β-lactamase Urinary Tract Infections

**Figure:**
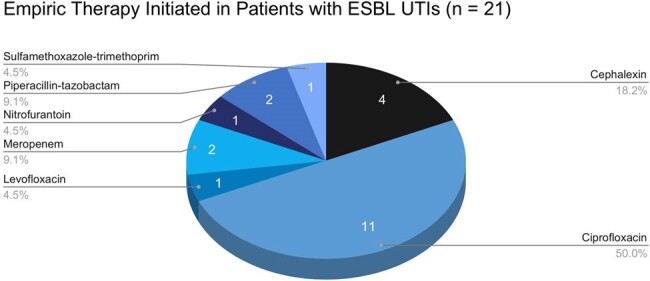

**Conclusion:**

The incidence of index UTI due to ESBL-producing bacteria is increasing while the UTI rate remains consistent, creating the need to identify optimal treatment and prevention strategies in KTR.

Secondary Outcome: Total Urinary Tract Infections in the First Year Post-Transplant

**Figure:**
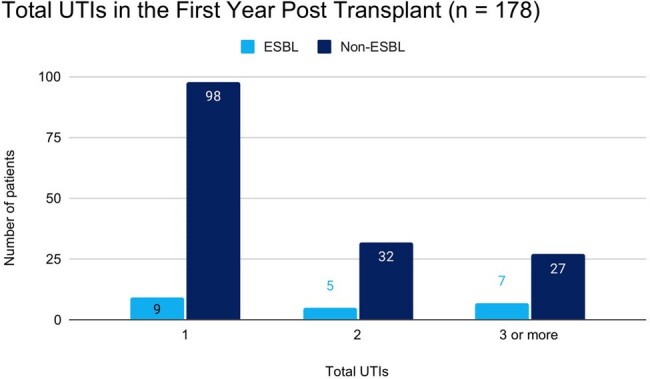

**Disclosures:**

All Authors: No reported disclosures

